# Large-scale mining disease comorbidity relationships from post-market drug adverse events surveillance data

**DOI:** 10.1186/s12859-018-2468-8

**Published:** 2018-12-28

**Authors:** Chunlei Zheng, Rong Xu

**Affiliations:** Department of Population and Quantitative Health Sciences, School of Medicine, Case Western Reserve University, 2103 Cornell Road, Cleveland, 44106 OH USA

**Keywords:** FAERS, Association rule mining, Disease comorbidity network

## Abstract

**Background:**

Systems approaches in studying disease relationship have wide applications in biomedical discovery, such as disease mechanism understanding and drug discovery. The FDA Adverse Event Reporting System (FAERS) contains rich information about patient diseases, medications, drug adverse events and demographics of 17 million case reports. Here, we explored this data resource to mine disease comorbidity relationships using association rule mining algorithm and constructed a disease comorbidity network.

**Results:**

We constructed a disease comorbidity network with 1059 disease nodes and 12,608 edges using association rule mining of FAERS (14,157 rules). We evaluated the performance of comorbidity mining from FAERS using known disease comorbidities of multiple sclerosis (MS), psoriasis and obesity that represent rare, moderate and common disease respectively. Comorbidities of MS, obesity and psoriasis obtained from our network achieved precisions of 58.6%, 73.7%, 56.2% and recalls 87.5%, 69.2% and 72.7% separately. We performed comparative analysis of the disease comorbidity network with disease semantic network, disease genetic network and disease treatment network. We showed that (1) disease comorbidity clusters exhibit significantly higher semantic similarity than random network (0.18 vs 0.10); (2) disease comorbidity clusters share significantly more genes (0.46 vs 0.06); and (3) disease comorbidity clusters share significantly more drugs (0.64 vs 0.17). Finally, we demonstrated that the disease comorbidity network has potential in uncovering novel disease relationships using asthma as a case study.

**Conclusions:**

Our study presented the first comprehensive attempt to build a disease comorbidity network from FDA Adverse Event Reporting System. This network shows well correlated with disease semantic similarity, disease genetics and disease treatment, which has great potential in disease genetics prediction and drug discovery.

## Background

Analysis of disease relationship plays an important role in understanding disease biology and discovering new drug treatments [1]. Disease similarity often indicates underling genetic connections. For example, genetic loci for complex diseases have been identified by examining the association between Mendelian diseases and complex diseases [2]. Studying disease relationship is an important strategy for disease gene discovery [3, 4]. Disease relationship is also widely used in drug discovery. For example, discovery of shared genetics of psoriasis and multiple sclerosis led to dimethyl fumarate, an anti-psoriasis drug, to be used in treatment of relapsing-remitting multiple sclerosis [5]. In addition, many drug repurposing strategies are based on disease relationship [6].

Several disease relationship networks have been reported. Some of them are based on phenotypic similarity, such as disease manifestation network (DMN) [7], clinical trial [8], and some are genetics based, such as human disease network (HDN) derived from Online Mendelian Inheritance in Man (OMIM) [9] and complex disease network (CDN) derived from genome-wide association studies (GWAS) [10]. In addition, comorbidity-based disease networks have also been constructed. Rhetsky et al. developed a statistical model to estimate the co-occurrence relationship for each pair of 160 diseases and demonstrated that comorbidities are genetically linked [11]. Park et al. and Hidalgo et al. detected the comorbidity pairs from the Medicare claims with statistical measures [12, 13]. Roque et al. mined pairwise disease correlations using similar measures from medical records of a psychiatric hospital [14]. All these networks provided valuable information about disease relationship. However, these studies have limitations. For example, Park’s study focused on elder patients aged 65 years or older and patients in Roque’s study were only from a single health center.

FDA Adverse Event Reporting System (FAERS) contains adverse event reports from manufacturers, consumers and healthcare professionals for all marketed drug and therapeutic biologic products [15]. FAERS is a large-scale database that contains patient diseases, medications, drug adverse events, demographics of around 17 million case reports, which has been extensively explored for detecting drug safety issues. But the rich information of FAERS is still not systematically mined for disease comorbidity. Recently, we used association rule mining algorithm to reveal the link of colorectal cancer with obesity, which demonstrated the feasibility of this method in disease comorbidity study [16, 17]. Multiple disease comorbidities are common in clinic setting [18] and the advantage of association rule mining is that it can flexibly detect multiple disease comorbidity [17]. In this study, we use association rule mining of FAERS to obtain the disease co-occurrence patterns and then constructed a disease comorbidity network (DCN) based on mined association rules.

To our best knowledge, this is the first comprehensive effort in constructing a large-scale disease comorbidity network from 17 million case reports available in FAERS. Through comparative analysis, we demonstrated that disease comorbidity network accurately captures disease-disease relationships published in the literature and has great potential in both disease genetics prediction and drug discovery.

## Methods

**Fig. 1:**
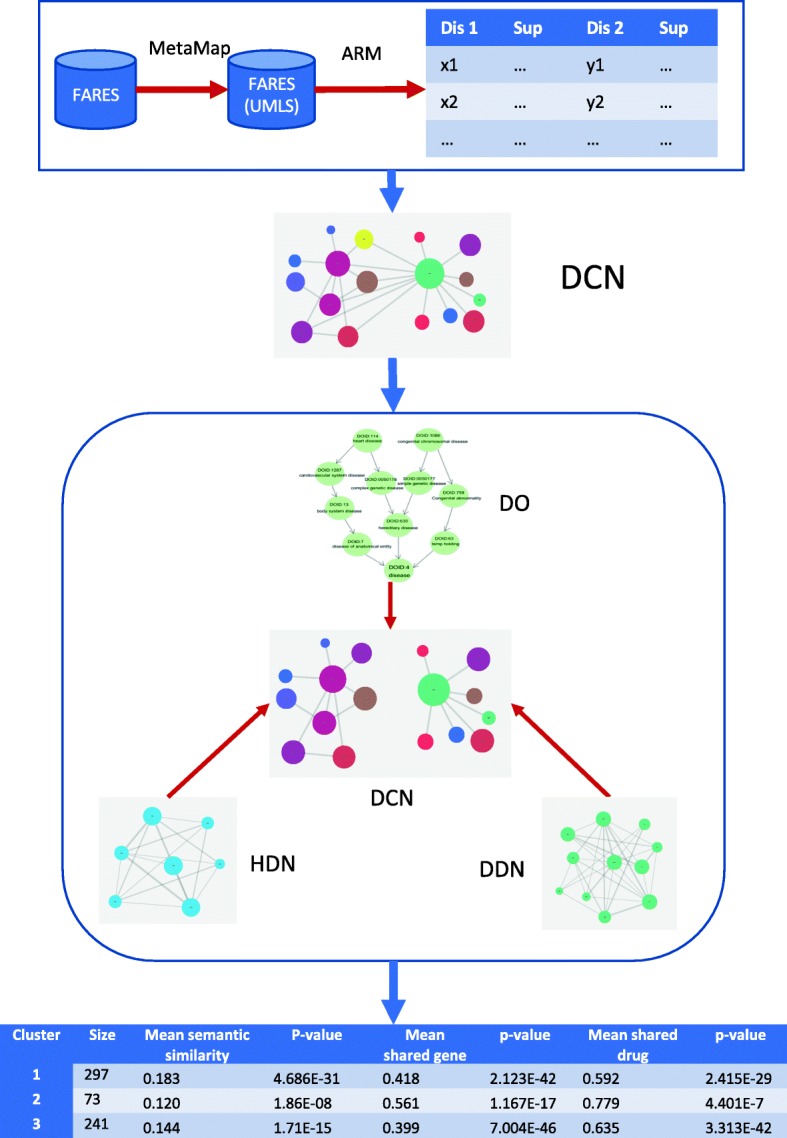
Diagram for our overall methods. ARM: association rule mining; DCN: disease comorbidity network; DO: disease oncology; HDN: human disease network (GWAS); DDN: disease drug network

The overall approach in this study includes three steps (Fig. 1). We firstly used association rule mining of FAERS indication data to obtain disease comorbidity patterns. Secondly, we constructed a disease comorbidity network (DCN) based on mined rules. Thirdly, we detected the inherent clusters of DCN and examined its correlation with disease genetics, semantic similarity and drug treatment.

### Datasets

FAERS data was downloaded from US Food and Drug Administration (FDA) [15], which contains 17,305,542 case reports for indications from 2004 to 2017. Disease genetic data were extracted from US National Human Genome Research Institute (NHGRI) [19]. The GWAS catalog contains 34,790 disease-gene associations for 14,062 genes and 1665 common complex diseases/traits. Drug-disease associations were extracted from biomedical literature [20–22], which contains 9216 drug-disease pairs for 1483 drugs and 1381 diseases. Disease ontology was downloaded from The OBO Foundry [23], which contains 10,903 disease terms.

### Construction of a disease comorbidity network (DCN) by association rule mining of FAERS

FAERS from 2014 to 2017 was used in this study to explore disease comorbidity patterns. After removing reports with unknown indications, the data contained 6,480,372 case reports and represented 15, 721 indications of drugs. In order to facilitate downstream analysis, we mapped indications (represented as MedDRA terms) into 12 semantic types that are classified into disorders in UMLS using MetaMap (2016 V2 release) [24]. 12 semantic types of disorders include Acquired Abnormality, Anatomical Abnormality, Cell or Molecular Dysfunction, Congenital Abnormality, Disease or Syndrome, Experimental Model of Disease, Finding, Injury or Poisoning, Mental or Behavioral Dysfunction, Neoplastic Process, Pathologic Function and Sign or Symptom. 12,225 of 15,721 (77.76%) indications were mapped. The clean data contained 6211 diseases and 5,784,501 case reports.

We then applied Frequent Pattern (FP)-growth algorithm (implemented in Weka) [25, 26] into this data to obtain the disease co-occurrance patterns, which is a list of rules between two sets of diseases, represented in the form {X->Y}, for example, {anxiety, diabetes mellitus -> multiple sclerosis}. FP-growth is a widely used association rule mining algorithm based on FP-tree data structure. Choosing proper support and confidence is a trade-off between precision and recall of disease comorbidities. Here, support > 12 and confidence > 0.5 were used according to performance of validation diseases. Total 14,157 rules were obtained. We constructed an undirected and unweighted disease comorbidity network based on these rules, in which nodes are all diseases at both sides and edges are established between each pair of diseases in both sides.

### Clustering of DCN

We used Girvan-Newman algorithm [27, 28] to detect communities in this disease comorbidity network. Girvan-Newman algorithm is based on edge betweenness and edge with biggest betweenness is removed in each iteration. Number of communities of a network depends on how many edges are removed. Modularity metric is computed in each iteration and optimized communities are obtained by maximizing the modularity of network [27].

### Correlation analysis of disease comorbidity network with disease genetic network

We constructed a weighted human genetic network (HDN) based on genome wide association data, in which diseases are represented as nodes and edge is added if two diseases share common genes. Edge weight represents number of share genes between them. Based on genetic information in HDN, we firstly calculated pairwise shared genes in each cluster of DCN obtained from community detection (see above section). Shared genes of a cluster C(G) is defined as average of pairwise shared genes in Eq. 1:

1 $$  C(G) = \frac{1}{m} \sum\limits_{d1\neq d2,d1,d2\in D}g(d1,d2)  $$

where *d*1,*d*2 are pairwise diseases in cluster, *g*(*d*1,*d*2) is shared genes between *d*1 and *d*2, *D* is disease node set in each cluster and m is number of total pairwise diseases in each cluster.

### Correlation analysis of disease comorbidity network with disease treatment network

For computation of shared drugs in each cluster of DCN, we firstly constructed of a disease drug network (DDN) based on FDA drug label data and biomedical literature. Then pairwise shared drugs in a cluster were calculated based on this DDN and share drugs of a cluster C(D) is defined as the average of pairwise shared drugs in Eq. 2:

2 $$  C(D) = \frac{1}{m} \sum\limits_{d1\neq d2,d1,d2\in D}d(d1,d2)  $$

where *d*1,*d*2 are pairwise diseases in a cluster, *d*(*d*1,*d*2) is shared drugs between *d*1 and *d*2, *D* is disease node set in each cluster and m is number of total pairwise diseases in each cluster.

### Correlation analysis of disease comorbidity network with disease semantic network

Disease ontology was used for computing semantic similarity of pairwise disease in each cluster of DCN, which is defined as:

$$sim(d1,d2)= \max_{a\in A(d1,d2)} - \log p(a)$$

where *A*(*d*1,*d*2)is the set of common ancestors for *d*1 and *d*2. Semantic similarity of a cluster *C*(*SIM*) is computed as average of pairwise disease semantic similarity in Eq. 3:

3 $$  C(SIM) = \frac{1}{m} \sum\limits_{d1\neq d2,d1,d2\in D}sim(d1,d2)  $$

where *d*1,*d*2 are pairwise diseases in a cluster, *sim*(*d*1,*d*2) is semantic similarity between *d*1 and *d*2, *D* is disease node set in each cluster and m is number of total pairwise diseases in each cluster.

Random network was built as a network with the same network structure but nodes are randomly shuffled. We generated 100 random networks and computed the shared genes, shared drugs and semantic similarity in each cluster for each network. T-test was used to compute the significance of each cluster compared with corresponding random networks.

### Prioritization of diseases associated with Asthma

In our case study, we used asthma as the seed and random walk with restart (RWR) to rank the diseases that associated with Asthma in DCN. RWR is a ranking algorithm that has been used to prioritize disease genes [29]. Ranking result is expressed as a probability vector at steady state, representing the probability of each node can be reached from the seed. Assuming *p*_0_ is the seed vector, *p*_*k*+1_, the probability vector at k + 1 step, is defined in Eq. 4:

4 $$  p_{k+1} = (1 - \gamma) M p_{k} + \gamma p_{0}  $$

where *γ* is the restart probability rate and M is adjacency matrix of DCN. *γ* is set to 0.15. Loop stopped when |*p*_*k*+1_−*p*_*k*_|<10^−6^, indicating probability vector is stable.

## Results

### Properties of Disease Comorbidity Network

**Fig. 2:**
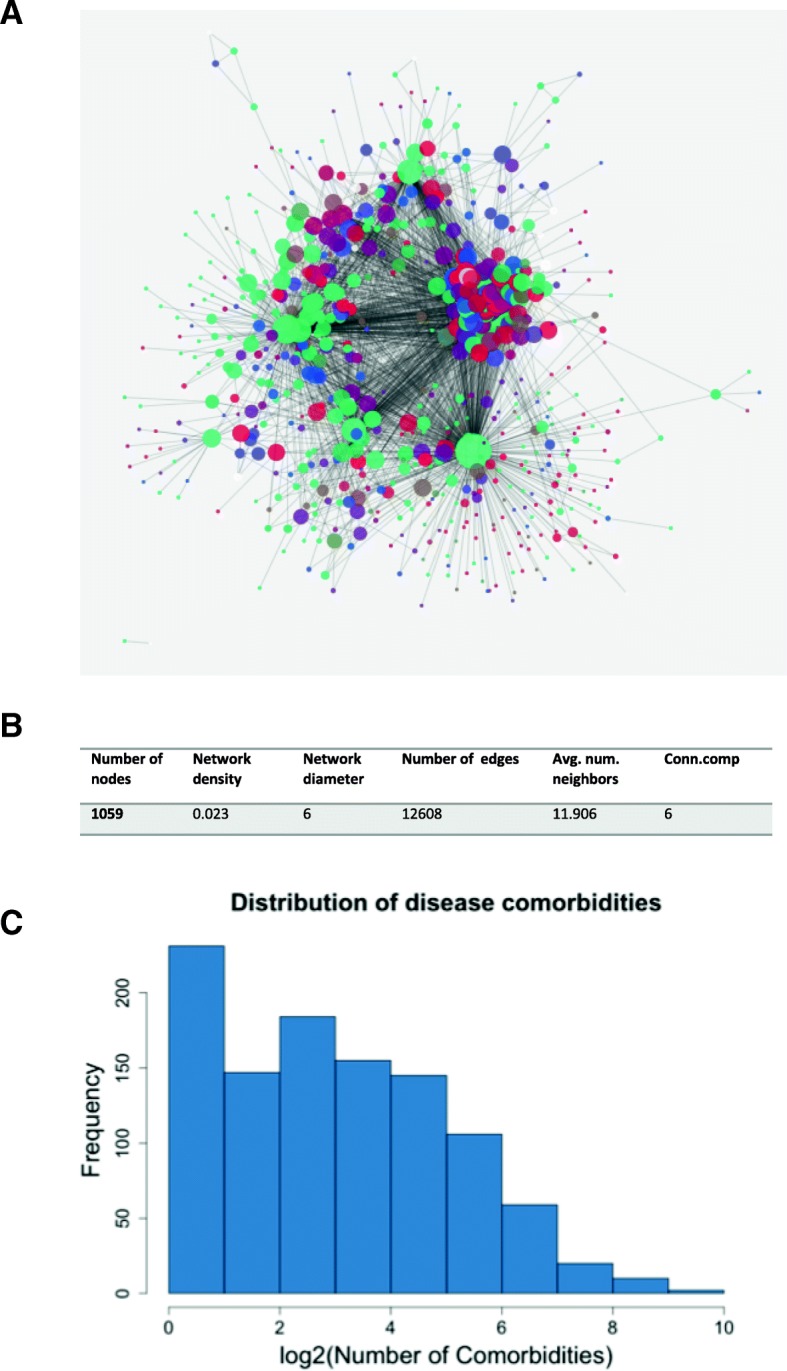
Characteristics of disease comorbidity network. **a** Visualization of DCN. Diseases are represented as nodes and the size of each node is proportional to the degree of that node. Node color represents disorder class (SOC in MedDRA) to which it belongs. Edges between nodes are represented as the co-occurrence of diseases. **b** Basic network property of DCN. **c** Distribution of disease comorbidity in DCN

Based on association rules from large-scale mining of FAERS, we constructed a disease comorbidity network (DCN), which contains 1059 nodes and 12,608 edges (Fig. 2a). This network is relatively sparse with density of 0.023 (Fig. 2b). Node degrees, i.e., comorbidities for each disease, range from 1 to 685, with median of 7 (Fig. 2c). The nodes with large degrees represent common comorbidities that co-occur with many diseases, such as hypertension, diabetes, depression, anxiety, etc.

Using Girvan-Newman community detection algorithm, DCN can be grouped into 6 clusters with more than 10 nodes (Fig. 2a). We further classified each disease into one of 27 system organ classes (SOC) based on Medical Dictionary for Regulatory Activities (MedDRA), represented as different node colors in the graph. To verity if these clusters reflect inherent disease associations, we computed the top enriched diseases in each cluster (Table 1). We can see that each cluster represents the specific types of diseases. We also noticed that the different types of diseases are grouped together, which reflects the additional level of disease associations.

**Table 1:** Top enriched diseases in each cluster

Cluster	Enriched diseases	Folds	*P* value
1	Cardiac disorders	3.72	3.08e-07
	Vascular disorders	2.28	1.46e-04
2	Metabolism and nutrition disorders	3.17	3.11e-05
3	Psychiatric disorders	8.71	3.33e-33
4	Renal and urinary disorders	2.63	2.06e-02
	Musculoskeletal and connective tissue disorders	2.54	1.48e-02
5	Eye disorders	10.38	3.56e-03
6	Infections and infestations	5.19	2.57e-03

### Evaluate disease comorbidity mining using three diseases: multiple sclerosis, psoriasis and obesity

Disease comorbidity is a complicated and dynamic concept and no systematic database is available, which makes evaluation of our disease comorbidity network difficult. Here, we evaluated DCN by comparison with literature reports, especially using information from corresponding national health organizations. Three diseases, including multiple sclerosis (MS), psoriasis and obesity were chosen for this purpose, which represent rare, moderate and common disease separately. Multiple sclerosis (MS) is a demyelinating disease in which the insulating covers of nerve cells in the brain and spinal cord are damaged. It is estimated that 2.3 million people have MS worldwide. Psoriasis is a chronic inflammatory disorder associated with significant morbidity and mortality. The prevalence of psoriasis among US adults ages 20 years and older is 3.2% [30]. Obesity is a medical condition in which excess body fat has accumulated to the extent that it may have a negative effect on health. More than one-third (36.5%) of U.S. adults have obesity [31]. Table 2 lists comorbidities of MS, psoriasis and obesity. Comorbidities of MS were extracted from National Multiple Sclerosis Society [32] and a literature report [33]; Comorbidities of psoriasis were obtained from National Psoriasis Foundation [34]; Comorbidities of obesity were obtained from Centers for Disease Control and Prevention [35].

**Table 2:** Comorbidities for obesity, psoriasis and multiple sclerosis

Disease	Comorbidities
Obesity	Hypertension, Coronary heart disease, Stroke, Dyslipidemia, Type 2 diabetes,
	Gallbladder disease, Osteoarthritis, Sleep apnea and breathing problems,
	Some cancers (endometrial, breast, colon, kidney, gallbladder and liver),
	Mental illness (depression, anxiety)
Psoriasis	Cardiac event, Stroke, Crohn’s Disease, Diabetes, Metabolic syndrome, Obesity,
	Osteoporosis, Uveitis and Liver Disease, Cancer, Depression
Multiple Sclerosis	Spasticity, Bladder disorder, Bowel problem, Vision problem, Fatigue, Weakness,
	Chronic lung disease, Hypertension, Diabetes, Numbness, Sexual problem, Pain,
	Headache, Epilepsy, Cognitive problem, Seizure, Tremor,
	Psychiatric problems (depression, bipolar disorder, anxiety and schizophrenia)

**Fig. 3:**
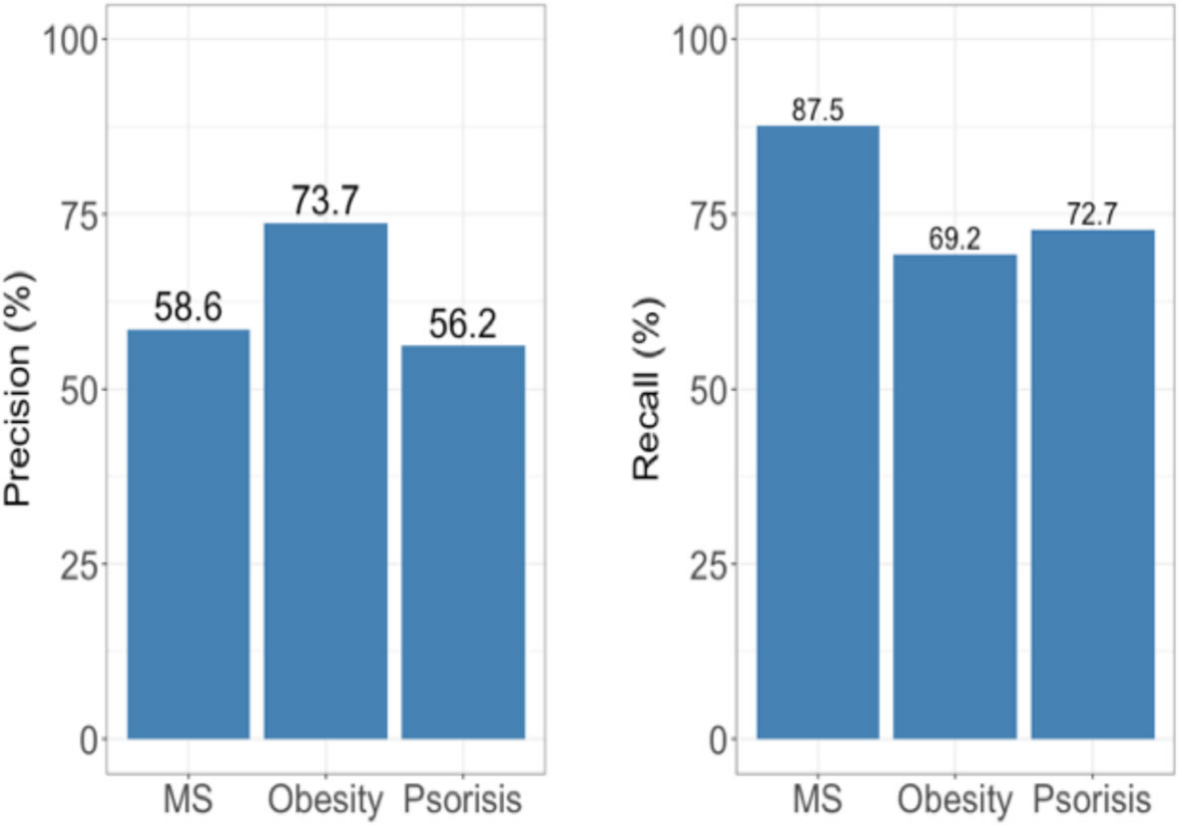
Precision and recall of DCN for MS, obesity and psoriasis

In DCN network, we considered all its neighbor nodes as comorbidities of given disease. To test the performance of our network, we extracted the comorbidities of these three diseases from DCN and compared with literature report as mentioned above. DCN achieved precision of 58.6%, 73.7% and 56.2%, and recall of 87.5%, 69.2% and 72.7% for MS, obesity and psoriasis separetely (Fig. 3).

### Disease comorbidity network significantly correlates with disease semantic similarity

Semantic similarity is a measurement that calculates the disease distance based on disease ontology. High semantic similarity between two diseases indicates that they share more pathological processes. Pairwise disease semantic similarity in each cluster was computed as mentioned in method and cluster semantic similarity is the average of all pairwise disease similarities in that cluster. Compared with random networks, semantic similarity in each cluster of DCN is significantly higher (Table 3).

**Table 3:** Statistics for semantic similarity of disease comorbidity network

Cluster	Size	Semantic similarity (DCN)	Mean semantic similarity (Random)	*p*-value
1	297	0.183	0.109	4.686E-31
2	73	0.120	0.095	1.860E-08
3	241	0.144	0.109	1.710E-15
4	335	0.131	0.110	2.048E-10
5	63	0.322	0.085	9.080E-92
6	50	0.200	0.097	5.273E-65

### Disease comorbidity network significantly correlates with disease genetics

Disease comorbidities often have common genetic causes and common phenotypic features. To test if our network essentially captures this observation, we computed the shared genes in each cluster and compared with random networks. Shared genes in pairwise diseases were obtained from disease-gene network constructed using GWAS resources. Overall shared genes in each cluster is the average of shared genes in all disease pairs in that cluster. The result showed that each cluster of DCN shares significantly more genes than that in random networks, indicating that DCN captures genetic relationship between diseases (Table 4).

**Table 4:** Statistics for shared genes of disease comorbidity network

Cluster	Size	Shared gene (DCN)	Mean shared gene (Random)	*p*-value
1	297	0.418	0.061	2.123E-42
2	73	0.561	0.012	1.167E-17
3	241	0.399	0.015	7.004E-46
4	335	0.433	0.053	5.733E-34
5	63	0.534	0.009	3.520E-18
6	50	0.413	0.029	1.836E-18

### Disease comorbidity network significantly correlates with disease drug treatment

Furthermore, we investigated if DCN captures disease drug treatment information. Similar method was used to compute shared drugs in each disease comorbidity cluster, we used disease-drug network to obtain shared drugs between two diseases. Compared with random networks, shared drugs in each cluster are also significantly higher (Table 5). Taken together, these correlation analyses demonstrate that our disease comorbidity network essentially captures disease-disease relationship.

**Table 5:** Statistics for shared drugs of disease comorbidity network

Cluster	Size	Shared drug (DCN)	Mean shared drug (Random)	*p*-value
1	297	0.592	0.136	2.415E-29
2	73	0.779	0.178	4.401E-7
3	241	0.635	0.062	3.313E-42
4	335	0.542	0.070	1.999E-36
5	63	0.610	0.180	4.149E-6
6	50	0.678	0.110	2.394E-5

### Disease comorbidity network reveals interesting disease associations/comorbidities - a case study

To demonstrate that DCN can be used for discovering novel disease relationship, we use asthma as an example. Asthma is a common long-term inflammatory disease of the airways of the lungs. Asthma is still an incurable disease. Main purpose of current medical treatments is to control symptoms. Many comorbidities of asthma have been observed, such as hay fever, allergy, obesity, sleep apnea, anxiety, depression, chest pain and cough, which makes asthma more difficult to control. Nevertheless, some diseases, such as hypertension and cardiovascular disease, shows association with asthma, but is still in controversial [36–38]. Therefore, understanding comorbidities of asthma is important for disease management and underlying biology.

We used random walk with restart to find diseases that are associated with asthma. Asthma was used as the seed and we ranked the other diseases in DCN, which represents the probabilities of each disease can be reached. We expected that comorbidities of asthma should rank high since they generally share common genetics. We can see that all of them rank in top 7% except obesity (Table 6), which further demonstrated the robust and usefulness of our network. Interestingly, hypertension and cardiovascular diseases, including atrial fibrillation, cardiac failure and stroke, also ranked very high (Table 6), suggesting that they might be also closely related to asthma. Indeed, two recent studies supports our observation. One is a large sleep cohort study that demonstrated that late-onset asthma significantly increases cardiovascular diseases [39]. Another is a case-control study from Kaiser Permanente database that established hypertension is a comorbidity of asthma [40]. These evidences strongly demonstrate that our disease comorbidity network is able to reliably capture disease-disease relationship and have great potential to reveal novel disease relationships.

**Table 6:** Ranks of asthma associated diseases from disease comorbidity network

Disease	Rank (%)	Comorbidity
Hay fever (Allergic rhinitis)	2.55	Yes
Allergy	4.25	Yes
Obesity	22.10	Yes
Obstructive sleep apnea	6.61	Yes
Anxiety	0.66	Yes
depression	0.57	Yes
chest pain	1.79	Yes
cough	3.68	Yes
hypertension	0.00	No
atrial fibrillation	0.09	No
cardiac failure	1.51	No
stroke	2.17	No

## Discussion

We constructed a disease comorbidity network by association rule mining of large-scale post market surveillance database. This network is able to accurately capture disease comorbidities of literature reported and well correlated with disease semantic similarity, disease genetics and drug treatments. More interestingly, it can also discover associated diseases in debate due to inconsistent reports in literature, which can be explained by large-scale of FAERS dataset. All these properties of DCN indicate that it has a great potential to be used in disease genetics prediction and drug discovery. We note that the way FAERS data is collected may bias the network as compared to the general population. Further evaluation of these biases will be required in subsequent work.

Previously, we also constructed a disease manifestation network (DMN) based on UMLS [7], which has been successfully used for disease gene prediction and drug discovery [17]. In addition, other disease-disease network based on electronic health record [14] and Medicare [12] are also available. Different data sources contain redundant and complementary information about disease relationship. We prospect that integration of these data sources may provide more power in disease gene discovery and drug repurposing.

## Conclusions

We built a comprehensive disease comorbidity network from FAERS using association rule mining. This network not only effectively retrieves known comorbidities of given disease, but is capable to reveal new disease-disease associations. Additionally, correlation analysis also shows that it reflects inherent disease relationships. This work provides a new source for study of disease genetics and drug discovery.

## Abbreviations

DCN: Disease comorbidity network
DDN: Disease drug network
FAERS: FDA adverse event reporting system
FP: Frequent pattern
GWAS: Genome-wide association studies
HDN: Human disease network
MedDRA: Medical dictionary for regulatory activities
MS: Multiple sclerosis
OMIM: Online mendelian inheritance in man
RWR: Random walk with restart
SOC: System organ classes
